# Expansion of human and animal sporotrichosis in Manaus, Amazonas State, Brazil

**DOI:** 10.1590/0102-311XEN180024

**Published:** 2025-06-27

**Authors:** Rosiane Mendes Valente, João Victor de Melo Verçosa, Eduardo Rodrigues de Souza, Natalia Prata Gordiano, Layssa do Carmo Barroso, Sonia Maria da Silva Carvalho, Marla Jalene Alves, Fernanda Rodrigues Fonseca, Maria Eduarda Grisolia, Maria Edilene Martins de Almeida, Paula Taquita Serra, Luis Andre Morais Mariúba, Ani Beatriz Jackisch-Matsuura

**Affiliations:** 1 Instituto Leônidas e Maria Deane, Fundação Oswaldo Cruz, Manaus, Brasil.; 2 Universidade Federal do Amazonas, Manaus, Brasil.; 3 Secretaria Municipal de Saúde de Manaus, Manaus, Brasil.; 4 Fundação de Vigilância em Saúde do Amazonas - Dra. Rosemary Costa Pinto, Manaus, Brasil.

**Keywords:** Sporothrix, Sporotrichosis, Epidemiologic Methods, Sporothrix, Esporotricose, Métodos Epidemiológicos, Sporothrix, Esporotricosis, Métodos Epidemiológicos

## Abstract

Sporotrichosis is an emerging fungal infection that has been causing concern in Brazil since the late 1990s and is the most prevalent and globally distributed among implantation mycoses. In Brazil, the increase in sporotrichosis cases is strongly associated with infections in cats. This study aimed to identify the epidemiological profile and expansion of sporotrichosis in the city of Manaus, Amazonas State, Brazil, based on cases reported from 2020 to 2023. Secondary data from confirmed sporotrichosis cases during this period in Manaus were employed. Human data were extracted from the Brazilian Information System for Notificable Diseases, the Manaus Municipal Health Department, and the RedCap database of the Amazonas Health Surveillance Foundation - Registry of Positive Cases. The animal data were extracted from the spreadsheet of the Dr. Carlos Durand Zoonoses Control Center in Manaus. Data analysis was performed using the RStudio software for the spatial distribution of cases in the QGIS program, which was used to define the areas with the highest absolute number of cases. From August 2020 to December 2023, 4,301 sporotrichosis cases were confirmed, including 3,403 animal cases (of which 99.6% involved cats) and 898 human cases. From 2022 to 2023, the number of human cases doubled and animal cases quadrupled, with cases reported across all areas of Manaus. The expansion of sporotrichosis was observed over a two-year period for nearly the entire area of Manaus, with a significant increase in cases and an association between animal and human cases.

## Resumo

Esporotricose é uma infecção fúngica emergente que causa preocupação no Brasil desde o final dos anos 1990, sendo a micose subcutânea mais prevalente e amplamente distribuída. No Brasil, o aumento dos casos de esporotricose está fortemente associado a infecções em gatos. Este estudo teve como objetivo identificar o perfil epidemiológico e a expansão da esporotricose no município de Manaus, Estado do Amazonas, Brasil, com base em casos notificados entre 2020 e 2023. Foram utilizados dados secundários de casos confirmados de esporotricose durante esse período em Manaus. Dados sobre humanos foram extraídos do Sistema de Informação de Agravos de Notificação, da Secretaria Municipal de Saúde de Manaus e do banco de dados RedCap da Fundação de Vigilância em Saúde do Amazonas - Registro de Casos Positivos. Dados sobre animais foram extraídos da planilha do Centro de Controle de Zoonoses Dr. Carlos Durand em Manaus. Os dados foram analisados utilizando o software RStudio para a distribuição espacial dos casos no programa QGIS, que foi empregado para definir áreas com maior número absoluto de casos. De agosto de 2020 a dezembro de 2023, foram confirmados 4.301 casos de esporotricose, sendo 3.403 casos em animais (dos quais 99,6% envolveram gatos) e 898 casos humanos. De 2022 a 2023, o número de casos em humanos dobrou e os casos em animais quadruplicaram, com registros de casos em todas as áreas de Manaus. A expansão da esporotricose foi observada ao longo de dois anos em quase toda a área de Manaus, com aumento significativo de casos e associação entre casos em animais e humanos.

## Resumen

La esporotricosis es una infección fúngica emergente que ha causado preocupación en Brasil desde finales de los años 1990, por lo cual es la micosis subcutánea más prevalente y distribuida ampliamente. El aumento de casos de esporotricosis en Brasil está asociado estrechamente con las infecciones en gatos. Este estudio tuvo como objetivo identificar el perfil epidemiológico y el aumento de la esporotricosis en la ciudad de Manaos, estado del Amazonas, Brasil, a partir de los casos reportados entre 2020 y 2023. Se utilizaron datos secundarios de casos confirmados de esporotricosis durante este período en Manaos. Los datos sobre humanos se obtuvieron en el Sistema de Información de Enfermedades Notificables, en la Secretaría Municipal de Salud de Manaos y en la base de datos RedCap de la Fundación de Vigilancia Sanitaria del Amazonas -Registro de Casos Positivos. Los datos de los animales se obtuvieron de la hoja de cálculo del Centro de Control de Zoonosis Dr. Carlos Durand en Manaos. Los datos se analizaron utilizando el software RStudio para la distribución espacial de casos en el programa QGIS, que se utilizó para definir las áreas con el mayor número absoluto de casos. De agosto de 2020 a diciembre de 2023, se confirmaron 4.301 casos de esporotricosis, 3.403 casos en animales (de los cuales el 99,6% fueron en gatos) y 898 casos en humanos. De 2022 a 2023, el número de casos en humanos se duplicó, y los casos en animales se cuadruplicaron, con registros de casos en todas las áreas de Manaos. La propagación de la esporotricosis se observó a lo largo de dos años en casi toda el área de Manaos, con un aumento significativo de casos y asociación entre casos en animales y humanos.

## Introduction

Sporotrichosis is an emerging fungal infection that has been causing concern in Brazil since the late 1990s and its expansion is increasing worldwide ^1^. In November 2020, the World Health Organization (WHO) included sporotrichosis in its list of neglected diseases in the document approved at the 73rd World Health Assembly ^2^.

*Sporothrix* is a fungus belonging to the phylum Ascomycota, class Pyrenomycetes, order Ophiostomatales, and family Ophiostomataceae ^3^ ^,^ ^4^. In total, five main species of *Sporothrix* cause sporotrichosis in humans: *Sporothrix schenckii*, *Sporothrix brasiliensis*, *Sporothrix globosa*, *Sporothrix mexicana*, and *Sporothrix luriei*. However, the expansion of sporotrichosis in Brazil is associated with cat cases, in which the main etiological agent is *S. brasiliensis* ^5^ ^,^ ^6^.

Sporotrichosis is a subacute to chronic suppurative and granulomatous disease. Symptoms vary according to the form of the disease, ranging from a localized cutaneous infection to a systemic disease, with lesions and ulcers in various parts of the body, including internal areas such as joints, bones, eyes, the lymphocutaneous system, the lungs, and the central nervous system ^7^. 

To our knowledge, no reports of spontaneous resolution of sporotrichosis exist. The recommended drug treatment for the disease includes itraconazole, potassium iodide, and amphotericin B ^7^. The laboratory diagnosis of sporotrichosis is performed using various techniques, such as culture growth, histological staining, polymerase chain reaction (PCR), and sequencing, in addition to novel techniques such as immunoassays based on antigen-antibody interaction ^7^.

Outbreaks of sporotrichosis have been reported in Brazil for more than 25 years; however, in recent years, the fungus has spread to all regions of the country and neighboring countries ^1^ ^,^ ^6^ ^,^ ^8^ ^,^ ^9^. Recently, in the state of Amazonas, sporotrichosis has become an important public health issue, although records of this fungal infection date back to the beginning of the last century ^10^. The number of feline and human sporotrichosis cases has been increasing in Amazonas since August 2020. In 2021, sporotrichosis was classified as a disease of municipal interest in Manaus and was integrated into the list of notifiable diseases for the state of Amazonas and, subsequently, case notifications were initiated ^11^.

Understanding the geographical distribution and conducting epidemiological surveillance are crucial to guide measures of prevention and treatment, as well as to evaluate and respond to sporotrichosis trends worldwide, as the fungus may evolve over time. This approach can aid predict and avoid new epidemics ^12^. Therefore, this study aims to identify the epidemiological profile and expansion of sporotrichosis in Manaus using cases recorded from 2020 to 2023. 

## Materials and methods

### Study design and study area

**Figure 1:**
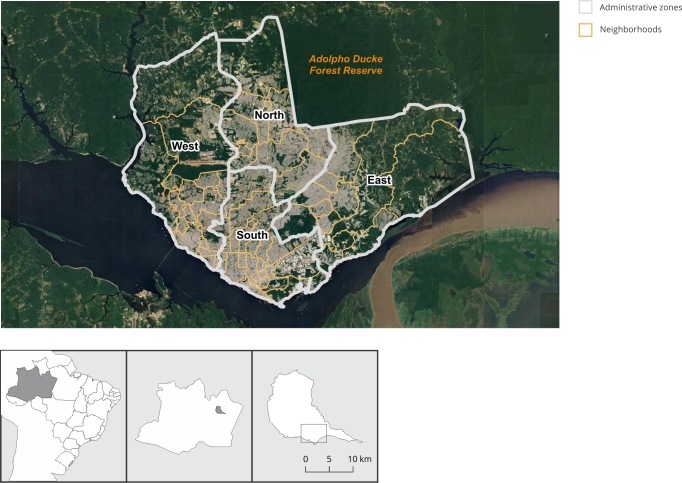
Study area: the city of Manaus, Amazonas State, Brazil. Map showing the urban areas of the municipality with administrative divisions.

Source: Brazilian Institute of Geography and Statistics ^28^. Image from Google Earth (https://earth.google.com/).

This is an ecological study that employs a spatial analysis and adopts a quantitative, descriptive, and exploratory approach using secondary data from confirmed sporotrichosis cases from 2020 to 2023 in the city of Manaus, capital of the state of Amazonas in Northern Brazil (Figure 1). Manaus shows a resident population of 2,063,689, with 11,401.092km^2^ of territorial area and 181.01 inhabitants per km^2^ ^13^. 

Data were obtained from confirmed cases of human sporotrichosis reported in health units in Manaus and from cases of animals with suspected sporotrichosis that were seen at the Dr. Carlos Durand Zoonoses Control Center (GECCZ, acroym in Portuguese) from August 2020 to December 31, 2023. The data collection period was chosen because it represents the beginning of reported cases in the municipality.

Human data were extracted from the Brazilian Information System for Notificable Diseases (SINAN, acronym in Portuguese), the Manaus Municipal Health Department (SEMSA, acronym in Portuguese) (sporotrichosis monitoring spreadsheets of the Department of Surveillance, Epidemiology, Environment, Zoonoses, and Occupational Health - DVAE, acronym in Portuguese), and the RedCap database implemented in October 2022 (in accordance with the Joint Technical Note n. 32/2022/SES-AM/FVS-RCP/FUHAM/FMT-HVD ^14^). Animal data were extracted from the Care Control and Animal Monitoring spreadsheet of the GECCZ Manaus.

The data were organized in a Microsoft Excel 2016 (https://products.office.com/) spreadsheet and analyzed using RStudio software (https://rstudio.com/), version 4.41. The spatial distribution of animal and human cases was analyzed in QGIS (https://qgis.org/en/site/), version 3.34, to identify areas with the highest absolute number of sporotrichosis cases and compare their distribution from 2020 to 2023. The analysis also included temporal trends to evaluate the progression of cases over time and identify patterns and areas of higher incidence. The study variables were categorized into general, clinical, epidemiological, and animal-related data.

General variables, applied to both humans and animals, included the year of diagnosis (2020 to 2023), final case classification (confirmed), place of residence, probable place of infection (neighborhood), administrative region (northern, southern, eastern, or western), and sex. Additionally, diagnostic confirmation relied on laboratory methods (mycological and cytological cultures) and clinical-epidemiological criteria. Cases with negative result in the laboratory examination and without sample collection, yet considered positive for sporotrichosis, had their diagnosis based on clinical and epidemiological criteria.

Clinical and epidemiological variables for human cases included lesion type (e.g., lymphocutaneous, fixed cutaneous, and extracutaneous), mode of transmission (e.g., scratching, bites, contact with skin or mucosal lesions, indirect or unknown contact), lesion characteristics (e.g., ulcers, nodules, papules), lesion site (e.g., hands, upper limbs, lower limbs, chest, head, etc.), demographic information (age group, ethnicity/skin color, schooling level), and urban or rural classification of neighborhoods within administrative zones. 

Animal-related variables included species (cats or dogs), sex, castration status, street access classification (e.g., indoor, free-roaming, community-cared stray, stray), and clinical outcomes (euthanized or deceased). The spatial distribution of animal cases was analyzed according to neighborhood and administrative zone, along with the locations of case concentrations over time.

### Ethical aspects

This study was approved by the Research Ethics Committee of the Amazonas State University (CAAE 76564923.5.0000.5016) and by the Ethics Committee in the Animal Experimentation at the Federal University of Amazonas (CEUA 23105.049659/2023-96).

## Results

In the present study, a total of 4,779 records were collected, of which 4,301 were positive for sporotrichosis (Table 1). From 2020 to 2023, 3,776 consultations involving animals in Manaus suspected of having sporotrichosis were registered at the Zoonoses Control Center. Positive diagnoses were made using laboratory methods and clinical-epidemiological criteria, which resulted in 3,403 confirmed cases of animal sporotrichosis. Regarding human cases, 1,003 patients residing in Manaus were recorded in the system, of which 898 were confirmed cases, 98 were discarded, and seven lacked sufficient information.

**Table 1:** Clinical-epidemiological data of sporotrichosis cases in Manaus, Amazonas State, Brazil, 2020 to 2023.

Characteristic	2020	2021	2022	2023	Total
Animal sporotrichosis					3,403
Sex					
Female	0	26	127	717	870
Male	15	112	432	1,558	2,117
Not informed	1	24	81	310	416
Species					
Canine	0	0	0	15	15
Feline	16	162	640	2,570	3,388
Castration status					
Castrated	0	15	98	733	846
Not informed	13	108	379	823	1,323
Street access classification					
Indoor	0	58	402	814	1,274
Free-roaming	13	38	98	1,265	1,414
Stray	1	56	117	418	592
Community-cared stray	2	3	12	47	64
No information	0	7	11	41	59
Clinical outcomes					
Euthanized or deceased	10	50	125	561	746
Human sporotrichosis					898
Sex					
Female	2	37	147	361	547
Male	1	39	93	218	351
Mode of transmition					
Infected by a bite	0	5	34	80	119
Infected by a scratch	2	32	88	223	345
Infected by contact with an animal’s lesions	2	20	79	150	251
Others or not informed	0	21	43	45	109
Time elapsed between the first symptoms and the start of treatment (human cases)					
1-6 days before starting treatment	0	9	11	36	56
7-30 days before starting treatment	1	31	113	183	328
31-60 days before starting treatment	0	14	64	71	149
61-90 days before starting treatment	1	7	11	20	39
91-120 days before starting treatment	0	3	7	10	20
Over 121 days before starting treatment	0	2	8	5	15
Start of treatment not informed	1	10	26	254	291

Note: an indoor animal does not have access to the street without supervision; a free-roaming animal has access to the street; a stray animal does not have an owner and depends on charitable organizations for medical care; a community-cared stray animal has a community that takes care of it, either neighborhood residents or a non-governmental organization.

Of the positive animal cases, 3,388 (99.6%) involved cats and 15 (0.44%) involved dogs. Regarding the sex of the animal, 2,117 (62.2%) were male, 870 (25.6%) were female, and 416 (12.2%) did not have sex information. The owners reported that 846 (24.9%) of the animals were castrated; of these, 844 were felines (390 females and 454 males) and two were canines (one male and one female). The animals were classified as follows: free-roaming, 1,414 (41.6%); indoor, 1,274 (37.4%); community-cared stray, 64 (1.88%); and stray, 592 (17.4%). 

Among the total animals, 746 (21.9%) died or were euthanized, of which 745 (99.9%) were cats and one (0.1%) was a dog. The majority had street access (69.85%; n = 521), 223 (29.9%) were indoor animals, and two (0.27%) lacked this information. No data were found regarding the interval from symptom onset to treatment initiation or the duration of treatment until death in these 746 animals.

Human sporotrichosis data indicate 898 positive cases; however, due to insufficient information, only 847 cases included a description of the probable location where infection occurred. Most of these cases were distributed across urban neighborhoods, with only one case reported in a rural area - specifically in the region known as the left bank of the Negro River in 2023.

Regarding the sociodemographic characteristics of the human cases, 547 (60.9%) were women and 351 (39.1%) were men, with ages ranging from 1 to 87 years (mean age of 40 years old). In terms of schooling level, the highest number of cases occurred among individuals with complete high school education (27.7%; n = 249). The most prevalent ethnicity/skin color category was mixed-race, with 724 (80.6%) cases, followed by white (11.1%; n = 100). 

Infection in humans occurred via scratches caused by animals (38.4%; n = 345), contact with skin lesions on animals (28%; n = 251), animal bites (13.3%; n = 119), contact with mucosal lesions on animals (2.23%; n = 20) and unknown transmission routes (12.1%; n = 109). The predominant clinical lesions in human patients were ulcers (74.1%; n = 665), nodules (24.4%; n = 219) and papules (12.7%; n = 114). The impacted body areas included the upper limbs (43.4%; n = 390) - some involving the hands (28.2%; n = 253) - the lower limbs (24.7%; n = 222), the chest (5.12%; n = 46), the head (3.5%; n = 31), the feet (2.9%; n = 26), the neck (1.6%; n = 14), the abdomen (1.6%; n = 14), and other areas (2.8%; n = 25), such as the face, outer ear, eyelids, gluteal muscles, and lower back.

In 2020, the first animal cases were reported in the western zone of the city of Manaus, specifically in the neighborhoods of Glória, with seven cases (43.75%); São Raimundo, with six cases (37.75%); Redenção, with one case (6.25%); and Vila da Prata, with one case (6.25%). In the northern zone, only one (6.25%) isolated case was reported in the Cidade Nova neighborhood. In humans, three reports were found: two cases from the Alfredo da Matta Hospital Foundation of Dermatology and Venereology (in August in the São Raimundo neighborhood and in November in the Glória neighborhood) and one case at the Heitor Vieira Dourado Tropical Medicine Foundation (in September in the Glória neighborhood).

In 2021, records show that sporotrichosis expanded throughout the municipality, with a total of 162 animal cases and 76 human cases affecting all four administrative zones of Manaus. The highest concentration of cases in both study populations was found in the western zone, impacting nine neighborhoods, with 141 animal cases (87.04%) and 68 human cases (89.47%), with higher numbers in the neighborhoods Santo Antônio (53 animals and 30 humans) and São Jorge (27 animals and 15 humans). 

By 2022, a significant increase was noted, with 640 animal cases and 231 human cases. Notably, prior to this period, the peripheral regions of Manaus had reported few or no sporotrichosis cases. The upward trend continued in 2023, with a notable surge: 2,585 animal cases and 538 human cases. Despite this increase, the Vila Buriti neighborhood remained free of recorded cases in both human and animal populations.

**Figure 2:**
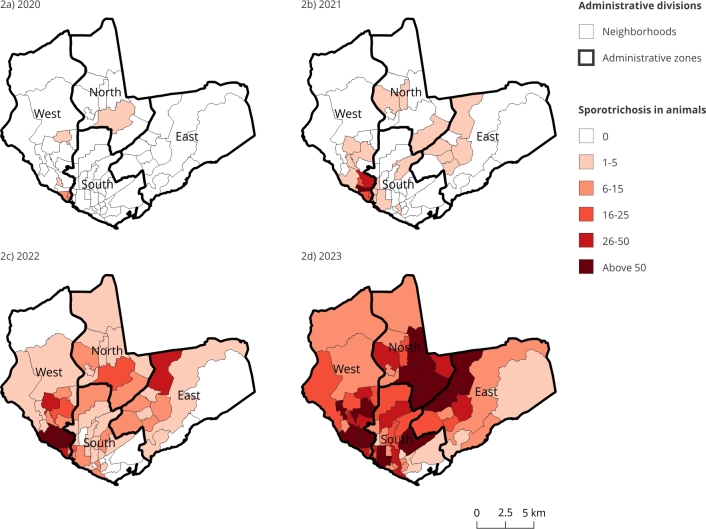
Spatial distribution of animal sporotrichosis cases in the neighborhoods of the administrative zones of Manaus, Amazonas State, Brazil, 2020 to 2023.

Source: Brazilian Institute of Geography and Statistics ^28^ and Manaus State Health Department ^20^.

**Figure 3:**
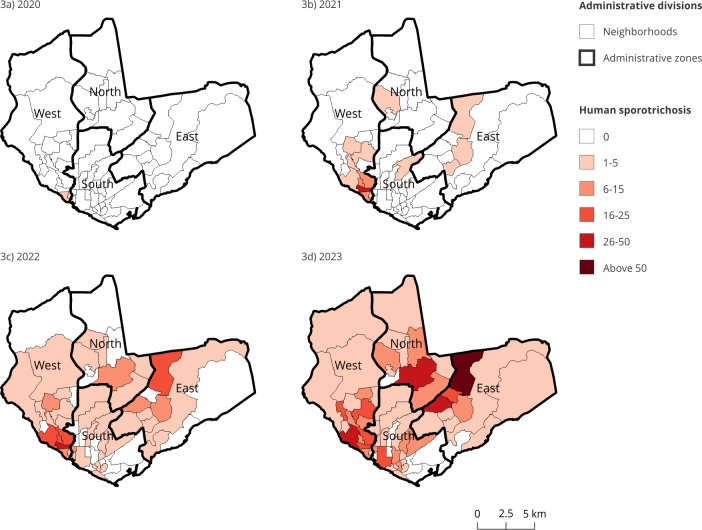
Spatial distribution of human sporotrichosis cases in the neighborhoods of the administrative zones of Manaus, Amazonas State, Brazil, 2020 to 2023.

Source: Brazilian Institute of Geography and Statistics ^28^ and Manaus State Health Department ^20^.

The spatial distribution of sporotrichosis cases in animals and humans across Manaus, as shown in Figures 2 and 3, highlights the progressive geographic spread of the disease. From 2020 to 2023, the disease expanded across the city, with cases initially concentrated in a few neighborhoods; then, by 2023, it had reached all administrative zones of Manaus.

## Discussion

Currently, the largest and longest-lasting outbreak of zoonotic sporotrichosis is associated with transmission by cats and has occurred in several geographical regions of Brazil. Zoonotic and epizootic transmission of *S. brasiliensis* began in the late 1990s in the state of Rio de Janeiro, Brazil, and is estimated to have impacted approximately 5,000 humans, 8,000 cats, and nearly 300 dogs ^6^ ^,^ ^15^. These data are probably underestimated because the notification of sporotrichosis cases is not mandatory in Brazil.

Animal and human sporotrichosis were first identified in Manaus in the second half of 2020. To control the disease, the health surveillance team at the SEMSA conducted an active search for suspected cases in animals and humans in the Glória neighborhood by visiting 474 households. Simultaneously, the GECCZ team performed clinical procedures on animals, including euthanasia, castration, and collection of samples for mycological examination in partnership with the Federal University of Amazonas (UFAM, acronym in Portuguese) ^16^. Euthanasia was performed when treatment was ineffective and the animal was suffering; the carcasses were submitted to incineration, carried out by a third-party service (Norte Ambiental Ltd.).

Regarding animal treatment, itraconazole (at dosages of 25, 50, and 100 mg formulated for animal usage) was distributed free of charge by GECCZ/SEMSA from April 2022, although with intervals. In 2023, distribution became consistent. Despite this important control measure, which was supplemented by other methods, such as public awareness campaigns promoting responsible pet ownership, animal castration programs to control stray populations, and improved sanitation practices in high-risk areas, animal contamination rates continued to grow exponentially. Antifungal drugs for the treatment of animal sporotrichosis are costly, and treatment lasts from three to six months, then maintained two to four weeks after clinical resolution ^6^. Therefore, treatment unavailability or discontinuity (influenced by abandonment) - a major barrier faced by health agencies in addressing the disease - has contributed to the spread of sporotrichosis in Manaus.

We highlight that sporotrichosis cases in Manaus occurred concomitantly with the COVID-19 pandemic that ravaged the world, during which health actions were directed to combat the disease. This focus led to delays in treatment for other diseases, especially those considered neglected tropical diseases (NTDs), such as fungal infections. Chaumont et al. ^17^ report that the prioritization of COVID-19 caused delays in health services such as the suspension of NTD programs, increased workload for health teams, and redirection of financial resources.

In December 2020, SEMSA Manaus released two technical notes warning of confirmed cases of sporotrichosis in the municipality of Manaus. The first, Technical Note n. 14/2020-DEVAE/SUBGS/SEMSA (2020, December 3rd) ^18^, provided guidance to health professionals, administrators, and workers in SEMSA’s services to immediately notify suspected cases and guided human investigations via the SINAN notification/conclusion form. The second case, Technical Note n. 001/2020-CCZ/DEVAE/SUBGS/SEMSA (2020, December 9th) ^19^, called for immediate notification by veterinary doctors attending and/or diagnosing animals with sporotrichosis.

In February 2021, sporotrichosis was included in the list of compulsory notifiable diseases (CND) via the approval of *Law n. 5,411* ^11^ by the State Legislative Assembly, mandating notification of animal and human sporotrichosis cases in public and private hospitals or veterinary clinics located in the state of Amazonas. 

Our study period indicates the first human sporotrichosis cases in August 2020; however, there are indications of underreporting. It should be noted that guidance regarding notification of human and animal sporotrichosis was provided to SEMSA Manaus health units, according to their responsibility for health. Only on October 20, 2022, was the Joint Technical Note n. 32/2022/SES-AM/FVS-RCP/FUHAM/FMT-HVD ^14^ released by the state health management department, which established the protocol for notification, diagnosis, clinical management, and epidemiological surveillance of human and animal sporotrichosis in Amazonas, providing guidance to health professionals and managers in public and private networks. On December 12, 2023, there was an update of the technical note, with the addition of SEMSA Manaus (Joint Technical Note n. 22/2023/FVS-RCP/FUHAM/FMT-HVD/SEMSA-MANAUS ^20^). 

Gremião et al. ^15^ emphasize that notification enables the public health system to monitor the disease, investigate and diagnose cases in a timely manner, record epidemiological data, and propose necessary interventions for disease management and control. Notification must be established throughout Brazil by the Ministry of Health to prevent disease spread without any epidemiological control.

Throughout the study period, animal sporotrichosis was disseminated across all administrative zones to a greater extent than human sporotrichosis. According to SEMSA Manaus, the disease spread in animals was attributed to several factors, including free-roaming cats that seek food over long distances, mating, territorial fights, and neglect or abandonment by owners, which leads to increases in the population of homeless animals ^16^. Andrade et al. ^21^ ^,^ ^22^ state that owned cats with street access are 2.54 times more likely to be infected with Sporothrix compared to those restricted to the home environment. 

The Glória neighborhood was the first in Manaus to report animal sporotrichosis in 2020, followed by the first human case in the São Raimundo neighborhood. These neighborhoods show similar characteristics, with infrastructure and basic urban sanitation; however, due to spontaneous occupation, some areas show irregular and unplanned housing, in addition to the occupation of the slopes and edges of creeks, which are considered areas of risk ^23^.

These neighborhoods have the presence of creeks in common. For example, the Mindu creek, which originates in the Adolpho Ducke Forest Reserve and flows into the São Raimundo neighborhood. In bordering neighborhoods, cases of animal and human sporotrichosis intensified. In this hydrographic scenario - with most impacted neighborhoods located in the western zone - poor sanitation and infrastructure may have contributed to the rapid increase of the disease in Manaus. Barros et al. ^1^ reported that cases of human, feline, and canine sporotrichosis from 1987 to 1998 in the city of Rio de Janeiro were concentrated in poorer peripheral areas.

Moreover, analysis of ethnicity/skin color data provides valuable insights into economic and social vulnerability. Individuals classified as mixed-race race/color group were involved in most cases, aligning with the demographic profile of Northern Brazil, where this group predominates ^13^. However, this predominance may also reflect deeper social inequalities, as Brazilian Institute of Geography and Statistics (IBGE, acronym in Portuguese) ^24^ highlights that populations identifying as black, mixed-race, or Indigenous are more economically vulnerable, with limited access to basic sanitation and health services, thus increasing their susceptibility to diseases such as sporotrichosis. Thus, understanding these sociodemographic patterns is critical for tailoring public health interventions and addressing the structural determinants of health disparities.

According to demographic data from IBGE ^24^, the most populous neighborhoods in Manaus are in the eastern, northern, and western regions, starting with Jorge Teixeira with 133,448 inhabitants, followed by Cidade Nova with 124,935 inhabitants, Compensa with 73,111 inhabitants, and Alvorada with 61,696 inhabitants. Notably, the first human cases of sporotrichosis in Manaus were identified in the western (São Raimundo and Glória) and northern (Cidade Nova) areas, which show the highest number of animal sporotrichosis cases and a potential for increased human cases due to being highly populated areas. 

Correlating the maps of animal and human infections (Figures 2 and 3), similarity is observed between them, such as the western zone (Tarumã-Açu neighborhood), the eastern zone (Puraquequara neighborhood), and Colônia Antônio Aleixo, areas characterized by lower economic status (Manaus, 2021) ^25^. In contrast, the southern zone is predominantly commercial and industrial.

A crucial aspect that must be considered is the proximity of impacted neighborhoods to the Adolfo Ducke Forest Reserve. This is an area composed of primary Brazilian Amazon forest of 100km^2^ managed by the Brazilian National Institute for Amazonian Research (INPA, acronym in Portuguese). Over the years, the urban area has expanded, reaching the boundaries of the reserve in both sides, which is still frequently raided by hunters of wild animals and individuals interested in extracting forest products (2020) ^26^. 

The reserve is located next to the Cidade de Deus neighborhood, and in 2022 and 2023, 34 animal sporotrichosis cases and six human cases were reported. Given this, there are concerns regarding the possible interaction between wild animals and domesticated or free-roaming animals in this region, as well as of soil contamination from deceased animals, leading to infection among wild animals. Marks & Duncan ^27^ report that the interaction between domestic or free-roaming and wild animals in native forests or urban areas represents a potential risk factor, as these animals can be injured, killed, or infected, resulting in the mutual transmission of pathogens.

A region where with no reported sporotrichosis cases is the Vila Buriti neighborhood. This neighborhood has some particularities, such as housing a smaller population compared to the other neighborhoods in Manaus, being on the shore of the Negro River, showing areas part of the Manaus Free Trade Zone, holding a naval base with military barracks, and presenting the CEASA port, which interconnects Manaus with other municipalities - including the beginning of the BR-319 highway linking Manaus to Porto Velho, the capital of Rondônia state. The absence of cases in this region may be related to the characteristics of the region, differing from other neighborhoods, or to underreporting. 

It is also worth noting that in the western part of Manaus, the Jornalista Phelippe Daou Bridge connects Manaus to other municipalities, such as Manacapuru, Novo Airão, Iranduba, and Cacau Pirera, facilitating daily migration of people and potentially promoting the spread of sporotrichosis to other regions. Although it is not necessary to identify the species causing sporotrichosis to initiate treatment ^6^, molecular identification is crucial for studying the transmission corridors between Amazonas and other outbreak areas in Brazil. 

Therefore, the spread of sporotrichosis over two years was observed across nearly all areas of Manaus, with a significant increase in cases and a strong association between animal and human infections. The challenge remains to contain the spread of the disease to the interior municipalities of Amazonas, which have less support than Manaus in facing a potential epidemic, and preventing contamination of the Amazon forest fauna.
